# Checklist of the Diptera families Acartophthalmidae, Canacidae (including Tethinidae), Carnidae and Milichiidae of Finland (Insecta)

**DOI:** 10.3897/zookeys.441.7144

**Published:** 2014-09-19

**Authors:** Jere Kahanpää

**Affiliations:** 1 Finnish Museum of Natural History, Zoology Unit, P.O. Box 17, FI–00014 University of Helsinki, Finland

**Keywords:** Checklist, Finland, Diptera, biodiversity, faunistics

## Abstract

A checklist of 29 species in the smaller carnoid families Acartophthalmidae, Canacidae, Carnidae and Milichiidae (Diptera) recorded from Finland is presented. Tethinidae are also included as a subfamily of Canacidae. *Phyllomyza tetragona* Hendel is removed from the list as no reliable records of this species within the post-1944 borders of Finland could be found.

## Introduction

The superfamily Carnoidea (*sensu* Buck 2006 but excluding Cryptochetidae, see Wiegmann et al. 2011) includes seven families. The largest by far is Chloropidae, treated in a separate article in this issue. Four of the remaining six are found in Finland: Acartophthalmidae, Canacidae, Carnidae and Milichiidae. The remaining two families, Inbiomyiidae and Australimyzidae, are respectively restricted to the Neotropical and Australasian Regions.

All four families are rather poorly studied in Finland. The presence of additional, even undescribed, species is very likely in Carnidae and Milichiidae. The identification of all species of *Meoneura* was verified from male genitalia during the preparation of this checklist with one exception: the male of *Meoneura elongella* (Zetterstedt, 1838) remains undescribed. It is the only North European *Meoneura* with black halteres and as such easily recognised.

World catalogues have recently been published for Canacidae (Munari and Mathis 2010), Carnidae (Brake 2011), and Milichiidae (Brake 2000). The Finnish species of these families were last listed in Hackman (1980).

**Table 1.** Number of species by family.

Family	Number of species in			Level of knowledge
	World (Pape et al. 2011)	Europe	Finland	
Acartophthalmidae	5	3	3	average
Canacidae	322	38	1	average
Carnidae	93 (Brake 2011)	39	13	poor
Milichiidae	278	43	12–13	average

## Checklist

suborder Brachycera Macquart, 1834

clade Eremoneura Lameere, 1906

clade Cyclorrhapha Brauer, 1863

infraorder Schizophora Becher, 1882

clade Muscaria Enderlein, 1936

parvorder Acalyptratae Macquart, 1835

superfamily Carnoidea Newman, 1834

**ACARTOPHTHALMIDAE** Czerny, 1928

***ACARTOPHTHALMUS*** Czerny, 1902

*Acartophthalmus bicolor* Oldenberg, 1910

*Acartophthalmus nigrinus* (Zetterstedt, 1848)

*Acartophthalmus pusio* Frey, 1947

**CANACIDAE** Jones, 1906

PELOMYIINAE Foster, 1976

***PELOMYIELLA*** Hendel, 1934

*Pelomyiella cinerella* (Haliday, 1837)

**CARNIDAE** Newman, 1834

***CARNUS*** Nitzsch, 1818

*Carnus hemapterus* Nitzsch, 1818

***MEONEURA*** Rondani, 1856

*Meoneura anceps* Frey, 1935

*Meoneura elongella* (Zetterstedt, 1838)

*Meoneura flavifacies* Collin, 1930

*Meoneura glaberrima* Becker, 1907

= *neglecta* Collin, 1930

*Meoneura lacteipennis* (Fallén, 1823)

*Meoneura lamellata* Collin, 1930

*Meoneura minutissima* (Zetterstedt, 1860)

*Meoneura neottiophila* Collin, 1930

*Meoneura obscurella* (Fallén, 1823)

*Meoneura prima* (Becker, 1903)

= *seducta* Collin, 1937

*Meoneura triangularis* Collin, 1930

*Meoneura vagans* (Fallén, 1823)

**MILICHIIDAE** Schiner, 1862

MADIZINAE Czerny, 1909

***DESMOMETOPA*** Loew, 1866

*Desmometopa m-nigrum* (Zetterstedt, 1848)

*Desmometopa sordida* (Fallén, 1820)

*Desmometopa varipalpis* Malloch, 1927

***LEPTOMETOPA*** Becker, 1903

*Leptometopa latipes* (Meigen, 1830)

***MADIZA*** Fallén, 1810

*Madiza glabra* Fallén, 1820

MILICHIINAE Schiner, 1862

***MILICHIA*** Meigen, 1830

*Milichia ludens* (Wahlberg, 1847)

PHYLLOMYZINAE Curran, 1934

***NEOPHYLLOMYZA*** Melander, 1913

*Neophyllomyza acyglossa* (Villeneuve, 1920)

***PHYLLOMYZA*** Fallén, 1810

? *Phyllomyza equitans* (Hendel, 1919)

*Phyllomyza formicae* Schmitz, 1923

*Phyllomyza longipalpis* (Schmitz, 1924)

*Phyllomyza rubricornis* Schmitz, 1923

*Phyllomyza securicornis* Fallén, 1823

*Phyllomyza silesiaca* (Duda, 1935)

## Excluded species

*Phyllomyza tetragona* Hendel, 1924 not found within present borders

*Tethina grisea* (Fallén, 1823) misidentification?

*Tethina illota* (Haliday, 1838)

## Notes

***Meoneura***. At least two additional, possibly undescribed species occur in Finland.

***Phyllomyza equitans* (Hendel, 1919)**. Only females have been found in Finland.

***Tethina grisea* (Fallén, 1823)** and ***Tethina illota* (Haliday, 1838)**. Munari and Mathis (2010) list two *Tethina* species from Finland. Krogerus (1932) is mentioned as the source for the *Tethina grisea* record; there is no listed source for the record of *Tethina illota*. An older version of the world catalogue (Mathis and Munari 1996) mentions only *Tethina grisea* from Finland. No Finnish specimens of these species could be found in the Finnish Museum of Natural History (MZH) where most of the material collected by Krogerus is kept, or in any other Finnish insect collection. Unfortunately the identifications in Krogerus (1932) are rather unreliable. It is possible that *Tethina* species occur in Finland, but none are included in this checklist because the evidence for their presence is, in my opinion, insufficient.
